# Diversity of Dicotyledenous-Infecting Geminiviruses and Their Associated DNA Molecules in Southern Africa, Including the South-West Indian Ocean Islands

**DOI:** 10.3390/v4091753

**Published:** 2012-09-24

**Authors:** Marie E. C. Rey, Joseph Ndunguru, Leigh C. Berrie, Maria Paximadis, Shaun Berry, Nurbibi Cossa, Valter N. Nuaila, Ken G. Mabasa, Natasha Abraham, Edward P. Rybicki, Darren Martin, Gerhard Pietersen, Lindy L. Esterhuizen

**Affiliations:** 1 School of Molecular and Cell Biology, University of the Witwatersrand, Private Bag 3, P.O. Box Wits, Johannesburg, 2050, South Africa; Email: leigh.berrie@nhls.ac.za (L.C.B.); paxim@nicd.ac.za (M.P.); shaun.berry@sugar.org.za (S.B.); nuailavav@gmail.com (V.N.N.); mabasak@arc.agric.za (K.G.M.); natashaabraham@gmail.com (N.A.); 2 Mikocheni Agricultural Research Institute, P.O. Box 6226, Dar es Salaam, Tanzania; Email: jndunguru2003@yahoo.co.uk; 3 National Institute for Communicable Diseases, Private Bag X4, Sandringham, Johannesburg, 2131, South Africa; 4 South African Sugarcane Research Institute, 170 Flanders Drive, Private Bag X02, Mount Edgecombe, 4300, South Africa; 5 The Institute of Agricultural Research of Mozambique, Av. Das FPLM, No. 269 C.P. 3658, Maputo, Mozambique; Email: nurbibicossa@gmail.com; 6 Biotechnology Center, Eduardo Mondlane University, Praca 25 de Junho. Caixa, Potal 257, Maputo, Mozambique; 7 Crop Protection and Diagnostic Center, ARC-Roodeplaat-VOPI, Private Bag X134, Pretoria, 0001, South Africa; 8 Institute of Infectious Disease and Molecular Medicine, University of Cape Town, Observatory, Cape Town, 7925, South Africa; Email: ed.rybicki@gmail.com (E.P.R.); darrenpatrickmartin@gmail.com (D.M.); 9 ARC-Plant Protection Research Institute and University of Pretoria, Private Bag X134, Pretoria, 0001, South Africa; Email: Gerhard.Pietersen@up.ac.za; 10 Department of Biochemistry, University of Johannesburg, PO Box 524, Auckland Park, 2006, Johannesburg, South Africa; Email: lesterhuizen@uj.ac.za

**Keywords:** Geminiviruses, sweepoviruses, dicotyledenous crops, southern Africa, eastern Africa, south-west Indian Oceans, diversity, recombination

## Abstract

The family *Geminiviridae* comprises a group of plant-infecting circular ssDNA viruses that severely constrain agricultural production throughout the temperate regions of the world, and are a particularly serious threat to food security in sub-Saharan Africa. While geminiviruses exhibit considerable diversity in terms of their nucleotide sequences, genome structures, host ranges and insect vectors, the best characterised and economically most important of these viruses are those in the genus *Begomovirus*. Whereas begomoviruses are generally considered to be either monopartite (one ssDNA component) or bipartite (two circular ssDNA components called DNA-A and DNA-B), many apparently monopartite begomoviruses are associated with additional subviral ssDNA satellite components, called alpha- (DNA-*α*s) or betasatellites (DNA-*β*s). Additionally, subgenomic molecules, also known as defective interfering (DIs) DNAs that are usually derived from the parent helper virus through deletions of parts of its genome, are also associated with bipartite and monopartite begomoviruses. The past three decades have witnessed the emergence and diversification of various new begomoviral species and associated DI DNAs, in southern Africa, East Africa, and proximal Indian Ocean islands, which today threaten important vegetable and commercial crops such as, tobacco, cassava, tomato, sweet potato, and beans. This review aims to describe what is known about these viruses and their impacts on sustainable production in this sensitive region of the world.

## 1. Introduction

Geminiviruses (family: *Geminiviridae*) in the genus *Begomovirus* are among the most devastating pathogens worldwide of a variety of cultivated crops, including cassava, sweet potato, beans, tomato, cotton and grain legumes [1,2,3,4,5,6,7,8]. Geminiviruses are distinct in having circular, single-stranded DNA (ssDNA) genomes that are encapsidated within twinned icosahedral virions [9]. Displaying substantial diversity in terms of their primary nucleotide sequences, genome structures, host ranges and insect vectors, the family *Geminiviridae* has been divided into four different genera. Besides the begomoviruses, these include the genera *Mastrevirus*, *Curtovirus*, and *Topocuvirus* [9]. The *Begomovirus* genus, with over one hundred and ninety two recognized species, contains more species than all the other geminivirus genera combined [9,10]. 

The primary species demarcation criterion for begomovirus classification is based on sequence similarity: to be classified as a new species a newly described DNA-A component (or DNA-A-like genome) must share less than 89% nucleotide (nt) identity with the DNA-A component of another previously recognised begomovirus species [9]. At higher taxonomic levels the begomoviruses can be subdivided into New World and Old World members. New World viruses nearly all have bipartite genomes (*i.e.*, with DNA-A and DNA-B genome components), with both components needed for infectivity [11]. In contrast, the majority of begomoviruses in the Old World apparently have monopartite genomes, and most of these interact with a class of ssDNA satellite molecules known as alpha- and betasatellites [12,13,14]. A small number of Old World begomoviruses, such as *Tomato yellow leaf curl virus* (TYLCV) [15,16] and *Tomato leaf curl virus* (ToLCV) [17], have true monopartite genomes containing only a DNA-A-like molecule that is sufficient to cause wild-type disease symptoms. Phylogenetically distinct from both Old World and New World begomoviruses are both a diverse group of bipartite begomoviruses infecting legumes (collectively called “legumoviruses”) which are largely restricted to Asia, and a group of monopartite begomoviruses infecting sweet potatoes (collectively called “sweepoviruses). 

All begomoviruses (including sweepoviruses and legumoviruses) are transmitted by the whitefly *Bemisia tabaci* (Gennadius) in a persistent, circulative manner to eudicotyledenous plants [18,19,20,21,22]. Worldwide, various begomovirus species are recognised as emergent threats to agriculture. Over the past three decades, the increasing threat posed by these viruses in a variety of cropping systems [1,23,24,25,26] has been linked to the global spread of ultra-invasive members of the *B. tabaci* species complex. The global emergence of begomoviruses is, however, likely attributable to a combination of additional factors including the innate evolutionary adaptability of begomoviruses to novel host and geographical ranges, agricultural intensification that has favoured rapid whitefly population expansion and the modern international trade in horticultural products that has spread host species, viruses and whitefly biotypes outside their natural geographical ranges [27,28]. 

Geminiviruses in general, but begomoviruses in particular, have the capacity to rapidly evolve via mutation and genetic recombination (involving both standard homologous recombination and component/satellite reassortment) [28,29,30,31,32,33,34]. It is through these processes of molecular diversification that widely distributed, normally weed-infecting begomoviruses, when transmitted into exotic cultivated host species (where either the virus has been transferred outside its natural environment or a new species has been introduced into the virus’ natural environment), have the capacity to rapidly adapt to these new host species. Adaptive potential can also enable host-range switching when viruses are transmitted to novel alternative hosts within their natural environments by whitefly types or species that feed on an unusually broad range of plant species (a characteristic associated with invasive whitefly biotypes). Eventually, this concerted begomovirus evolution can lead to the emergence of new species with altered pathogenic potential and expanded geographical and/or host ranges.

Besides viral adaptation, the capacity of *B. tabaci* to adapt to and exploit modern agricultural systems has also been of paramount importance in the emergence of novel begomoval plant diseases. *B. tabaci* is considered to constitute a cryptic species complex whose members are morphologically indistinguishable but exhibit a range of genetic, biological and behavioral variation [22,35,36,37,38] Population analysis using the mitochondrial cytochrome oxidase I (mtCOI) gene sequence as a molecular marker have revealed 24 cryptic species that generally group phylogenetically according to their current geographical ranges. The exceptions, to this rule are the invasive B and Q whitefly types that now have a near global distribution [38]. The extent to which *B. tabaci* populations vary genetically and biologically throughout sub-Saharan Africa has yet to be fully explored. Analysis of *B. tabaci* populations from nine African countries have revealed the existence of five endemic sub‑Saharan Africa subclades (SSAF-1 through -5), that are coexisting with the invasive B and Q types in a number of regions [39,40,41,42,43]. The majority of SSAF clade members associate with cassava [22,39,40,44,45,46,47,48,49,50] but some SSAF members have been documented to colonize indigenous plants and other vegetable crops [43,48,49,51,52]. The relevant importance of each of these *B. tabaci* types as begomovirus vectors are however dependent on their host associations, fecundity and virus transmission characteristics. The cassava associated types transmit at least seven begomovirus species to cassava [44,45,50,53] and the vegetable associated biotypes transmit a number of other different begomovirus species [43,48,49,51,52]. It is however, the extremely polyphagous B and Q types that are considered the most important vectors of emergent begomoviruses. As is mentioned before, the spread of these invasive types has coincided with the emergence of new begomovirus diseases in many different regions around the world, including sub-Saharan Africa [1,3,54,55,56].

In sub-Saharan Africa, many of these emergent begomoviruses cause huge economic losses for small and large scale farmers alike and can have a particularly dire impact on subsistence farmers. As is the case elsewhere in Africa, the maintenance of food security in the Southern African Community Development (SADC) countries [57] is an extremely pressing problem [58], with per capita food consumption having declined substantially over the past decade. As a result, the SADC region as a whole is a net importer of food with some countries also relying intermittently on food aid. Food insecurity in the SADC region is associated with high levels of poverty [58], with food shortages being caused in part by global fluctuations in agricultural commodity prices. One leading factor contributing to these fluctuations has been increased global demand over the past decade for sugar, maize, cassava and other crops as biofuel feeds. The SADC region is approximately the size of Europe and includes a variety of climatic zones and agro-ecosystems such that, in addition to it being suitable for cereal cultivation (cereals contribute to ~50% of African caloric intake), it is suitable for the cultivation of a wide variety of other crops including cassava, sweet potato and assorted fruits and vegetables. In order to achieve African food security in an era of rapid human population growth and looming global food shortages, it is imperative that Africa experiences accelerated economic growth, and achieves increased agricultural productivity. 

With the ever present threat of emerging and emergent begomovirus diseases in the SADC and SWIO regions, it is critically urgent that attention be focused on strategies to both stop ongoing losses to these viruses and prevent the future emergence of any further begomoviral diseases. Given that a wide range of factors from viral evolutionary dynamics (such as viral synergisms, rapid mutation, frequent recombination and reassortment) to vector evolutionary and population dynamics (such as polyphagous invasive whitefly biotypes) to changing human agricultural practices and movement dynamics (such as high intensity monoculture or human trafficking of infected material across borders) have likely contributed to the emergence of begomoviral diseases, it will be almost certainly be necessary to necessary to tackle this problem on multiple fronts [59,60,61,62,63,64]. Considering the present and future scale of emerging new geminiviral disease problems in important crops, it is unsettling that so little is currently known about the begomoviruses of the SADC and SWIO regions. The hosts in which these viruses have been identified include a relatively small number of introduced cultivated species such as cassava, tobacco, tomato, bean and sweet potato (Table 1). Virtually nothing is currently known about the diversity of begomoviruses in uncultivated indigenous African plant species. Also, whereas defective DNAs [65,66] have been identified in association with cassava mosaic viruses, nothing is known about the diversity and distribution of these epidemiologically important pathogenicity-modulating entities. This review therefore focuses on what little is known about the eudicot-infecting begomoviruses of the SADC and SWIO regions in the hope that it will spark increased study of this important group of present and future agricultural pests.

## 2. Diversity of Dicotyledenous-Infecting Begomoviruses in SADC Countries and South-West Indian Ocean (SWIO) Islands

Amongst the begomoviruses infecting crops in sub-Saharan Africa, the most important is probably the begomovirus disease complex affecting cassava [5,67]. Although cassava mosaic disease and its associated begomovirus (*Cassava latent virus*, now known as *African cassava mosaic virus*) have been reported numerous times over the past one hundred and twenty years [68,69,70], it has only been over the past two decades with improved molecular detection and differentiation techniques like ELSIA, PCR and genome sequencing, that the full diversity of African cassava infecting geminiviruses has become appreciated. Besides the discovery over the last fifteen years of multiple new cassava-infecting begomovirus species and major strain variants across the SADC and SWIO regions, there has concomitantly been the characterisation of a range of other begomovirus species infecting bean, tomato, tobacco and sweet potato [43,46,63,64,66,71,72,73,74,75,76,77,78,79,80,81,82,83,84,85,86,87,88,89]. Whereas begomovirus species complexes have been detected in tomatoes [63,81] and probably also occur in tobacco [90], it is presently unknown whether begomoviral diseases of these other species also involve similar complexes. In addition to the likely existence of a wide variety of economically relevant begomovirus species, small circular or defective DNA molecules associated with cassava- (South Africa and Tanzania) and tobacco- (Zimbabwe) infecting begomoviruses have also been reported [66,78,87]. These various begomovirus species, their major strain variants and associated DNA molecules are listed in Table 1. The sections below individually focus on each of the crop species affected by these viruses. Note that although bean yellow dwarf virus is a mastrevirus, it is the only known economically relevant eudicot-infecting non-begomoviral geminivirus in the SADC/SWIO region, and is considered here for the sake of completeness. 

### 2.1. Cassava (Manihot esculenta Crantz)

#### 2.1.1. General Introduction

Cassava (*Manihot esculenta* Crantz) is a perennial woody shrub of the *Euphorbiaceae* family and is a staple food for more than a billion people in about 105 countries, for which it provides an estimated one third of their caloric intake [91]. Cassava was introduced into Mozambique and Tanzania by the Portuguese in the 17th century and later spread westwards into South Africa and Swaziland [92]. Cultivation took hold only gradually and it appears that plantings in South Africa came mainly with the major tribal movements of the 1830s and 1860s [93]. In the SADC/SWIO regions, cassava is also extensively grown in Zambia, Malawi and Zimbabwe. Cassava was thought to be introduced into Zambia via the Congo basin (where it was well established by the early 1650s), and into Zimbabwe and Malawi via Portuguese trading routes from Mozambique on the east coast of Africa [94]. Cassava’s adaptability to adverse conditions such as marginal soils and erratic rainfall, and low maintenance requirements, makes it an ideal security crop for periods of drought and famine, especially for resource-limited subsistence farmers [95]. While cassava is becoming increasingly important in subsistence farming [95,96], its industrial use for starch and biofuel production is now rapidly increasing its global demand [97].

**Figure 1:**
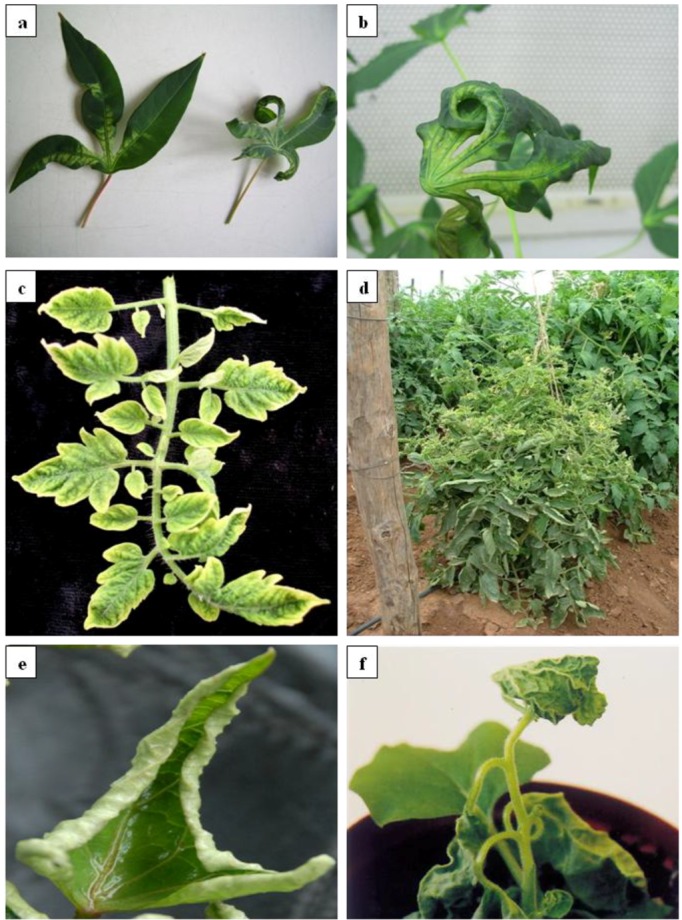
Symptom phenotypes of geminivirus-associated diseases caused by (**a**) *African cassava mosaic virus* (ACMV); (**b**) *South African cassava mosaic virus*-South Africa (SACMV-[ZA]); (**c**) and (**d**) *Tomato curly stunt virus* (ToCSV-[ZA:Ond:98]); (**e**) Sweet potato infected with a mixed infection of *Sweet potato mosaic associated virus* (SPMaV-[ZA:WP:2011]) and *Sweet potato leaf curl Sao Paulo virus* (SPLCSPV-[ZA;WP:2011]); f. *Tobacco leaf curl Zimbabwe virus* (TbLCZV-[ZW]).

Symptoms resembling those of cassava mosaic disease (CMD) were first reported by Warburg in 1894 [98] in what is now Tanzania. The disease was later reported in many other countries in east, west and central Africa and it is now known to occur in all the cassava-growing countries of Africa and the SWIO islands. Whereas in East Africa the disease was not reported to cause serious damage until the 1920s, in West Africa, CMD was first recorded in the coastal areas of Nigeria, Sierra Leone and Ghana in 1929 and had spread northward by 1945 [99]. The first epidemiological information on the disease came from a study by Storey and Nichols (1938) [69] who described virus strains based on the severity of disease they elicited, broadly dividing viruses into mild and severe strains. Storey and Nichols (1938) further described the mechanism of transmission and concluded that the whitefly *B. tabaci* was probably the vector. However the etiology of CMD was not clear until in the late 1970s when Bock and Guthrie (1978) [100] described a virus that could be transmitted by mechanical inoculation of sap from mosaic-infected cassava to *Nicotiana clevelandi* Grey. The causal agent of CMD was thereafter named *cassava latent virus*. The etiology of the virus was definitively determined in 1983 by Bock and Woods (1983) [101], who proved Koch’s postulates for the virus and named it *African cassava mosaic virus* (ACMV). Symptoms of this virus in cassava varied from mild mosaic to more severe symptoms of leaf curl (Figure 1a,b), leaf distortion, yellowing and plant stunting, depending on the infected cassava cultivar, the virus genotype(s) (mixed infections are common), and climatic conditions.

#### 2.1.2. Geographic Diversity of Begomoviral Species and Variants

**Figure 2:**
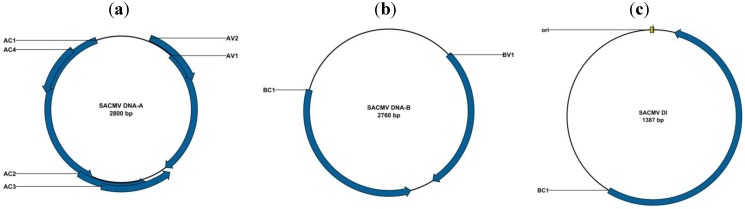
Genome organizations of (**a**) *South African cassava mosaic virus* (SACMV) DNA A component; (**b**) SACMV DNA B; (**c**) SACMV defective interfering molecule (DI).

CMD continues to be a major problem throughout sub-Saharan Africa [5,67,102] where it is now realised that at least seven distinct bipartite cassava mosaic begomovirus (CMB) species are associated with the disease. These species are *African cassava mosaic virus* (ACMV), *East African cassava mosaic virus* (EACMV), *East African cassava mosaic Cameroon virus* (EACMCV), *East African cassava mosaic Kenya virus* (EACMKV); *East African cassava mosaic Malawi virus* (EACMMV), *East African cassava mosaic Zanzibar virus* (EACMZV) and *South African cassava mosaic virus* (SACMV) [9,103] (Figure 2). The cassava-infecting bipartite begomoviruses cluster phylogenetically within either the African/SWIO OW begomovirus sub-clade (I) or the African/Cassava bipartite OW begomovirus sub-clade (IV) (Figure 2). Prior to 1994 only ACMV and EACMV were known to infect cassava in Africa and were thought to be limited to specific geographical areas, whereby ACMV was thought to occur only in West Africa and EACMV in East Africa [104]. However, improved diagnostic techniques such as PCR and rolling circle amplification (RCA) coupled with affordable sequencing have completely recharted the distributions of these and the many of the newly discovered CMBs [5,62,83,105]. To date, several studies have shown the presence of ACMV in all parts of the continent where cassava is grown and EACMV is now known to occur in West Africa as well. For example, Fondong *et al*. (2000) [61] isolated EACMCV-CM-[CM:98] in Cameroon, and more recently EACMV-[UG]was reported in Angola within the SADC region [106]. The occurrence of ACMV, EACMV, EACMMV and EACMCV species in several SADC countries such as Tanzania, Malawi and Zambia is also well documented [73,74,102,105]. Of the various CMBs, EACMV has so far demonstrated the highest genetic diversity due, at least in part, to the fact that EACMV variants have most frequently been the recombinational recipients of DNA-A and DNA-B fragments from other CMB species. Therefore, whereas over 56 variants or strains of EACMV have been reported [9], all ACMV variants that have so far been characterised from all over Africa have displayed substantially less diversity [5,61,62,83]. The extremely high diversity of EACMV in East Africa, suggests that this region may be the center of EACMV diversity and is possibly also the origin of the most economically relevant CMB species and strains [65].

Various new reports of CMBs in the SADC/SWIO region have emerged in the past decade. EACMV, SACMV-[MG;12] (Table 1) and ACMV are now known to occur in Madagascar [79] and recently, *Cassava mosaic Madagascar virus* (CMMGV), a new CMB species, was also discovered on this island [107]. DNA-A and -B shared the highest nucleotide sequence identity of 82% and 81.7%, respectively, with EACMZV-[KE:M-sa:K212:02] [107]. As mentioned above, EACMV and ACMV occur in Angola, often as mixed infections, and a recent survey in four provinces showed that whereas the severe EACMV-[UG] virus variant [102] likely has a widespread distribution in the country, ACMV was most prevalent (accounting for 85% of the CMB positive samples) [106]. Similarly, in the neighbouring Democratic Republic of Congo (DRC), EACMV-[UG] and ACMV have also been reported [108,109,110,111,112]. Interestingly, the cassava viruses in the north-eastern (Yamgambi province) part of Congo were also able to infect two leguminous species [108]. Together with EACMV and ACMV, SACMV occurs in the SADC countries South Africa, Swaziland, Mozambique, Zambia and Zimbabwe [80,112,113,114,115] and on Madagascar [79].

Although screening of southern African cassava samples for the presence of satellite DNAs has not been standard practice, in the South African and Mozambiquean studies where such screens were performed (using, for example universal beta satellite PCR primers described by Briddon *et al.* [116], no evidence of such satellites has been found. 

#### 2.1.3. Transmission and Diversity of Begomoviruses in South Africa and Mozambique

Although EACMV-UG and ACMV were detected in surveys, the only described CMB in South Africa remains SACMV, a virus that is transmitted by a South African whitefly population belonging to the so-called major sub-Saharan African (SSAF) cluster [39]. Sequence analysis indicates that the coat protein (CP) and common region (CR) of SACMV-[ZA] are most closely related to EACMV and the monopartite virus *Tomato yellow leaf curl virus* (TYLCV), respectively [76]. No recombination events have been detected in DNA-B of SACMV-[ZA]. The sub-Saharan Africa region harbours mainly non-invasive indigenous *B. tabaci* types that presumably serve as the natural vectors of the continent’s many indigenous begomovirus species [40,44]. These types cluster into five SSAF sub‑groups, named SSAF-1 through to 5 [42,43]. *B. tabaci* members within the SSAF major clade have been documented colonizing various vegetable crops and the majority also associate with cassava (*Manihot esculenta*) [22,39,40,44,45,46,47,48,49]. SSAF-1 is now known to be the most widely established group of whiteflies associated with cassava in Africa, and is likely the primary vector of the various CMB species that are distributed throughout the continent [9,39,40]. Whiteflies belonging to the SSAF-1 subclade are known to occur in eight African countries, including the SADC countries Zimbabwe, South Africa [39] and Mozambique [115]. The SSAF-2 subclade includes whiteflies from Uganda described previously as the invasive Uganda U2 population, that have been associated in the last two decades with extremely severe East African CMD epidemics [4,40,49]. One component of CMD epidemiology that is very different to that of many other geminiviruses is the role of humans in moving CMBs around the continent. Since cassava is propagated with cuttings, use of infected planting material is likely a major cause of losses to CMD throughout the continent. In South Africa, for example, it has been determined that approximately twice as many infections are caused via this route than are caused by whitefly transmission [114]. It remains almost completely unknown, what the natural host ranges of the various CMBs are, although geminiviruses have been detected in weed, ornamental and wild uncultivated crops. Small scale host range studies with CMBs such as SACMV‑[ZA] have indicated that they likely have narrow host ranges amongst other cultivated species such as *Nicotiana benthamiana*, *Nicotiana tabacum*, *Phaseolus vulgaris* and *Malva parviflora* [76]. The three major CMB species in the SADC and SWIO regions are ACMV, EACMV and SACMV, with ACMV the most prevalent virus and mixed EACMV:ACMV infections being relatively common. Although SADC whitefly populations associate with cassava and are capable of transmitting these CMB species, the main cause of CMD infections and the main driver of CMB movements is likely the widespread practice of cassava vegetative propagation using CMB infected cassava stakes.

#### 2.1.4. Recombination

Recombination amongst cassava begomoviruses on the African continent has been well documented [60,61,105]. In Mozambique, South Africa, the DRC and Angola, ACMV and EACMV have frequently been found together with one another within mixed infections indicating that there is a persistent danger that CMB recombinants with novel pathogenic properties might emerge within these countries [106,112,114,115]. The long-term maintenance of mixed CMB infections within vegetatively propagated cassava lines must surely create abundant opportunities for the evolution of synergistic interactions between the co-infecting viruses and for frequent recombination and genome component reassortments to occur. The introduction of ‘new’ viruses into an area and the presence of whiteflies can further exacerbate CMD spread by creating opportunities for mixed infections with resulting recombination/pseudo-recombination and synergism of the virus species involved. Pseudo‑recombination occurs when DNA-A of one virus trans-replicates with DNA-B of another [5]. It is likely, in fact, that many of the CMB species, including SACMV [76], EACMZV [117] EACMMV [60], and others [118] actually arose through recombination events arising from such mixed infections. Recently, further evidence of recombination between bipartite and monopartite begomoviruses, giving rise to the provisionally named *African cassava mosaic Burkino Faso virus*, was reported from Burkino Faso [118]. The minor parents were related to *Tomato leaf curl Cameroon virus* (FM210278) and *Cotton leaf curl Gezira virus* (FM210276). There is an ever present danger, therefore, that the next major CMD epidemic could be triggered by the emergence, from one of the countless millions of CMB mixed infections that likely exist throughout the SADC region, of a new ultra-pathogenic recombinant CMB species. From the perspective of CMD control management, information on sequence exchange patterns amongst cassava begomovirus complexes, and ecological interactions between environments virus, whitefly vector and host species, could be extremely valuable. 

#### 2.1.5. Defective Interfering DNAs Associated with CMBs

Subgenomic DNA molecules are often associated with geminiviruses [32,119,120,121] and are usually derived from deletions, insertion, duplication or rearrangement of their associated wild-type geminivirus genome and their satellites. These subgenomic defective interfering (DI) molecules are approximately half the size of their cognate helper virus and are packed in a viral coat protein [121,122]. Although DIs contains the intergenic region, found in all geminiviruses, they are dependent on the helper virus for replication, movement, and transmission [121]. DI molecules have been shown to ameliorate, delay and attenuate symptoms in plants infected with the helper virus [122,123]. It is suggested that DIs compete with the helper virus for essential host and viral factors, thereby inhibiting the replication of the helper virus leading to symptom amelioration [122,123,124]. Most DIs are derived from the DNA-B component of the helper virus, and the DIs derived from DNA-A are exclusively associated with monopartite geminiviruses with the exception of the recently discovered DNA-A derived DI of *East African cassava mosaic virus* (EACMV), a bipartite begomovirus [65]. DNA-B-derived DIs usually contain part of the ORF coding for the movement protein (MP) but the ORF coding for the nuclear shuttle protein (NSP) is completely absent. 

The first well-characterized DI from a geminivirus in sub-Saharan Africa was associated with DNA-B of *African cassava mosaic virus*-[Kenya] (ACMV-[KE]), in *N. benthamiana* [119]. To date, only four defective DNAs have been reported from crops in the SWIO/southern African regions, namely DNA-A derived DI (AY676464) of *East African cassava mosaic virus* (EACMV-[TZ15] (AY828226), a bipartite begomovirus in Tanzania [65]; a DNA-B derived DI associated with *South African cassava mosaic virus*-[South Africa:99] (accession number: JX233822) [66]; and two defective DNAs, namely ‘HG’ df DNA (AF368275) and ‘mild’ df DNA (AF368274), associated with the monopartite begomovirus TbLCZV-[ZW] from tobacco in Zimbabwe [78]. The DI (df DNA 15) derived from EACMV-[TZ15] DNA-A in Tanzania is 1,525 nt and has retained the *cis* elements for replication by the helper virus. The intergenic region and 5' region (±80%) of the AC1 Rep gene were retained. Biolistic inoculation of *Nicotiana benthamiana* with EACMCV and an infectious DI clone resulted in symptom amelioration, which correlated to an accumulation of DI and concomitant reduction in DNA-B levels infected leaves. Several studies have indicated that *de novo* DIs form more readily in model plants such as *N. benthamiana* compared to natural hosts [120,125]. Fewer DIs have been reported from naturally infected plants, but the EACMV-[TZ15] associated df DNA 15, SACMV-[ZA:99] associated DI DNA, and the TbLCZV-[ZW] were all isolated from field-infected cassava or tobacco, respectively. 

More recently, a defective interfering molecule (accession number: JX233822) has been associated with SACMV-[ZA] in a naturally infected field cassava plant collected from the Mpumalanga Province in South Africa [66] (Figure 2c). This DI is derived from SACMV-[ZA] DNA-B (Figure 2b), with the ORF BV1 (57 to 1,429 nt position) entirely deleted, and the carboxylic terminus of gene BC1 is deleted leaving only 82% of the BC1 gene intact [66]. Further studies indicated that no subgenomic particles were produced *de novo* when cassava plants were biolistically or agroinoculated with SACMV-[ZA] DNA-A and B infectious clones. An investigation to determine the effect of DI on SACMV-[ZA] replication and symptom development in an experimental host *N. benthamiana* and the cassava, the natural host, was performed [87]. Viral load (DNA-A and DNA-B) was accurately determined using quantitative real-time PCR, and symptoms were scored according to a symptom severity index (1–5) in plants agroinoculated with SACMV-[ZA] DNA-A and DNA-B infectious clones [76], or inoculated with SACMV-[ZA] + DI infectious dimer [126]. A direct measure of DI could not be established using quantitative real-time PCR due to 98% sequence homology between the DI and DNA-B of SACMV-[ZA]. However, the results from this study showed that the DI did reduce DNA-A and DNA-B viral titres in both *N. benthamiana* and cassava, despite symptoms persisting (albeit reduced in severity) over the time course of infection (28 and 42 dpi in *N. benthamiana* and cassava, respectively). What was interesting to note was that the impact of DI on reducing viral replication and symptom attenuation was different in the two plant systems. In cassava, the symptom attenuation was more pronounced compared with *N. benthamiana*, which correlated to the respective viral titres, while in *N. benthamiana* chlorosis, typical of SACMV-[ZA] infection, appeared to be reduced, with more leaf blistering and curling. This may be due to differences in viral-host factor interactions during viral replication between a model host (*Nicotiana benthamiana*) and a natural host (cassava) [127]. Cassava will have also co-adapted with SACMV-[ZA] over evolution and may have developed some tolerance to infection in contrast to *N. benthamiana*, a highly susceptible experimental host for multiple viruses [128]. Another significant observation was that titres of DNA-A and DNA-B showed a cyclic or modular pattern of increases and decreases during the infection process. Symptom severity did not appear to depend entirely on continuously decreasing pattern in levels of DNA-A or DNA-B over time in *N. benthamiana* and cassava, and a modular pattern of increases and decreases was observed from 7 to 28 dpi in tobacco, and 14 dpi and 42 dpi in cassava, for both SACMV-[ZA] infection alone, or co-inoculation with the DI dimer. Modulating increases and decreases in SACMV-[ZA] DNA-A and DNA-B titers in both plants systems may be a consequence of a cyclic replication pattern typical of a situation with bipartite viruses, such as SACMV-[ZA], where two DNA components are interdependent on each other for different functions at different times, such as replication (DNA-A) and movement proteins (DNA-B). Increases in DNA-A levels later on during the infection period indicate a recovery in DNA-A replication which may be predicted since DNA-A (AC1 and AC3 ORFs transcribe for the Rep-associated and replication enhancing proteins, respectively) is the rate-limiting factor for both its own autonomous replication and is required by both DNA-B and the DI for *trans*-replication [123,129]. Symptom amelioration suggests that efficient high replication of the DI by the helper virus is a prerequisite for symptom amelioration. However *trans-*replication and movement of DIs also requires high levels of cognate helper virus-encoded Rep and movement proteins, and therefore production of DNA-A and DNA-B components. Therefore symptom amelioration cannot only be due to selection, which may favour DIs out-competing the helper virus due to a size advantage. Relative spacial dynamics between helper virus and DI, not only within cells, but within whole leaves and the plant, are highly likely to play a significant role in the outcome of disease symptoms. Since DNA-A and B of SACMV-[ZA] compete for Rep binding in the common region, there is an ‘interdependence’ between these two components, which is likely to result in a modular pattern of increases and decreases in DNA-A in relation to DNA-B. The presence of an additional DNA-B-derived DI DNA is also likely to influence this process. Modulation of genomic and defective DI DNA components has also been observed in RNA helper and satellites [121]. However, despite interest in exploring the potential of dfDNAs for disease management, limitations of cognate helper virus specificity hamper broad adoption of this strategy to control begomovirus complexes.

### 2.2. Tomato

#### 2.2.1. General Introduction

The cultivated tomato (*Solanum lycopersicum*, formerly *Lycopersicon esculentum*) is an important vegetable crop with considerable nutritional and economic value. It belongs to the *Solanaceae* family, which in addition to *S. lycopersicum* L., contain more than 10 related wild species [130,131]. The cultivated tomato is thought to have originated in the New World, since the natural distribution of the wild species is restricted to the Andean region [132]. Domestication and use of tomato as food probably first occurred in Central America and was brought to Europe by the Spanish conquistadors in the sixteenth century and later introduced from Europe to southern and eastern Asia, Africa and the Middle East. Today, tomatoes are grown widely around the world, with more than 130 million tons of tomatoes at a value of over 30 billion dollars grown each year [91]. In the SADC and SWIO regions, tomatoes are commonly grown by subsistence and small-scale farmers and are one of the main vegetable crops sold by small-scale entrepreneurs in the informal economic sector. And estimated ~0.987 million tons of tomatoes are also grown commercially in this region with a total production area of ~ 60,000 hectares [91]. South Africa accounted for ~55% of this total, Tanzania for ~24%, the DRC for 5% and Madagascar for 4.1%.

Currently, begomoviruses are one of the most important and damaging threats to tomato production worldwide. During the last three decades, there has been a tremendous increase in the number of identified tomato infecting begomoviruses and presently there are more than 60 species many of which are components of six major begomoviral tomato disease complexes found in Africa, the Mediterranean, the Indian subcontinent, south east Asia, the Caribbean and South America. All of these species induce a disease characterized by varying degrees of stunting, compacted growth, leaf curling, leaf crumpling and leaf yellowing. The precise symptoms are known to vary between virus species (and in some cases between strains of the same species), with the specific tomato cultivar that is infected, the age of plants when infected, with environmental conditions and depending on the presence or absence of satellite molecules [1,61,133]. Infected plants normally show reduced fruit set and yield losses can reach as high as 100%, particularly when they are infected early in development [134]. The threat of emerging begomoviruses is also affecting tomato production in the SADC and associated SWIO islands. Since 1990 the presence of tomato infecting begomoviruses has been confirmed in eight of the countries/islands in these regions (South Africa, Tanzania, Comoros, Madagascar, Malawi, Mauritius and Reunion) but information regarding the impacts of tomato infecting begomoviruses in countries such as Angola, Botswana, the DRC, Namibia, Zambia and Zimbabwe is still lacking. Begomovirus infections of tomatoes were only confirmed in southern Africa for the first time as part of a survey to assess the global distribution of what is globally the most notorious begomoviral pathogen of tomatoes: *Tomato yellow leaf curl virus* (TYLCV).

#### 2.2.2. Geographic Diversity of Tomato Begomovirus Species and Variants

TYLCV causes tomato yellow leaf curl disease (TYLCD), which was first described in Israel in the 1930s, but probably first emerged in the vicinity of Oman or Iran in the early 1900s before moving to Israel in the 1920s [135]. It has since been transferred from Israel to the western Mediterranean region and onwards to the rest of the world where evidence exists for repeated introductions during the past three decades to Eastern Asia, the Americas and the SWIO island of Reunion [20,136,137]. On mainland Africa TYLCV-like disease was first diagnosed in Tanzania in 1990 [137,138] and Malawi in 1994 [139]. However, the serological methods used to make these identifications were not exacting enough permit species identification [140]. Although the genome sequence based identification of tomato infecting begomovirus species in the SADC and SWIO regions only began in earnest in the 2000s, by 1997 the first novel sub-Saharan tomato infecting begomovirus species was identified: *Tomato leaf curl Tanzania virus* (TYLCTV) [71]. TYLCTV likely caused an epidemic of yellow mottling, severe leaf curling and stunting symptoms in tomato plants grown the Makutupora district of Tanzania in 1997. In a subsequent Tanzanian survey of tomato infecting begomoviruses causing mild leaf curling and yellowing symptoms, a virus most closely related to the Mediterranean begomovirus species, *Tomato yellow leaf curl Sardinia virus* was identified [141]. Later full genome sequencing of the Tanzanian viral isolate from Arusha revealed it to be as a distinct monopartite begomovirus species called *Tomato leaf curl Arusha virus* (ToLCArV-[TZ:Ten:05]) [84]. 

At some time in the late 1980s or early 1990s, the Mld and IL strains of TYLCV spread from the western Mediterranean region (presumably human mediated means) to the islands of Reunion and Mauritius in the SWIO region [89,135,142]. While attempts to detect TYLCV on other SWIO islands have not detected this virus (for now TYLCV appears restricted in the SWIO region to Reunion), they have instead identified eleven novel tomato infecting apparently monopartite (evidenced for some of these species with infectious clones and suggested for all by the absence of any detectable DNA-B or satellite molecules) begomovirus species on the islands of Madagascar, and the Seychelles and Comoros archipelagos (Table 1) [63,81,82,143]. As is the case in the SADC region, little is known about the economic impacts of these viruses. 

The first report of a begomovirus infecting tomato plants in South Africa was from the Onderberg region in 1997 [86,144]. The affected tomato plants showed foliar symptoms similar to those induced by TYLCV, including upper leaf yellowing, reduction in leaflet area, upward curling margins, stunting and flower abortion (Figure 1c,d). Instead of finding TYLCV infecting these plants, however, a new monopartite species, called *Tomato curly stunt virus* (ToCSV-[ZA:Ond:98]) was identified [86,144]. Subsequent surveys have revealed that whereas ToCSV is the predominant tomato infecting begomovirus species in South Africa, there also exist at least three additional distinct monopartite begomovirus species tentatively named *Tomato curly stunt Mooketsi virus* (ToCSMV-[ZA:Mks:07]), *Tomato curly stunt Lanseria virus* (ToCSLV-[ZA:Lan:08]) and *Tomato curly stunt Noordoewer virus* (ToCSNV-[ZA:Nwr06:08]) [88,145]. While these other three species appear to have a more limited geographical range than ToCSV, they nevertheless cause symptoms similar to ToCSV in tomato plants. Although the economic cost of such infections is unknown, plants displaying what appear to be infection symptoms have been reported for many years, and as with TYLCV, infected plants can lose 95% or more of their yields such that these viruses could potentially become (if they have not already) a major constraint on tomato production in South Africa [86,145]. 

**Table 1:** List of mono- and bipartite geminiviruses and defective infecting subgenomic DNAs infecting dicotyledenous crops in southern and eastern Africa and south-west Indian Ocean Islands.

Geminivirus	Accession Number	Host	Location	Reference
*African cassava mosaic virus*				
African cassava mosaic virus-[Tanzania:2001] (ACMV-[TZ:01])	AY795982	Cassava	Tanzania	[83]
African cassava mosaic virus-[Tanzania:2001] (ACMV-[TZ:01])	AY795982	Cassava	Tanzania	[83]
African cassava mosaic virus-[Angola:2008] (ACMV-[An:08])	FJ807631	Cassava	Angola	Unpublished
African cassava mosaic virus-[Angola:AOS:2009] (ACMV-[An:09])	GU580897	Cassava	Angola	Unpublished
African cassava mosaic virus-[Congo:2008] (ACMV-[DRC:08])	FN435277	Cassava	Congo	[108]
African cassava mosaic virus-[Congo:2008] (ACMV-[DRC:08])	FN435275	Cassava	Congo	[108]
African cassava mosaic virus-[Congo:2008] (ACMV-[DRC:08])	FN435273	Cassava	Congo	[108]
African cassava mosaic virus-[Congo:2008] (ACMV-[DRC:08])	FN435271	Cassava	Congo	[108]
African cassava mosaic virus-[Congo:2008] (ACMV-[DRC:08])	FN435276	Cassava	Congo	[108]
African cassava mosaic virus-[Congo:2008] (ACMV-[DRC:08])	FN435274	Cassava	Congo	[108]
African cassava mosaic virus-[Congo:2008] (ACMV-[DRC:08])	FN435272	Cassava	Congo	[108]
African cassava mosaic virus-[Congo:2008] (ACMV-[DRC:08])	FN668378	Cassava	Congo	Unpublished
African cassava mosaic virus-[Congo:2008] (ACMV-[DRC:08])	FN668379	Cassava	Congo	Unpublished
*Bean yellow dwarf virus*				
Bean yellow dwarf virus-[South Africa:Mpumalanga:1994] (BeYDV-[ZA:Mpu:94])	Y11023	Bean	South Africa	[72]
*Cassava mosaic Madagascar virus*				
Cassava mosaic Madagascar virus-[Madagascar:Toliary:2006] (CMMGV-[MG:Tol:06])	HE617299		Madagascar	[107]
*Cotton leaf curl Gezira virus*				
Cotton leaf curl Gezira virus-[Madagascar: Fort Dauphin:2001] (CLCuGV-Be[MG:For:01])	AM701767	Bean	Madagascar	[63]
*East African cassava mosaic virus*				
East African cassava mosaic virus-[Congo:2008] (EACMV-[DRC:08])	FN435281	Cassava	Congo	[108]
East African cassava mosaic virus-[Congo:2008] (EACMV-[DRC:08])	FN435279	Cassava	Congo	[108]
East African cassava mosaic virus-[Congo:2008] (EACMV-[DRC:08])	FN435280	Cassava	Congo	[108]
East African cassava mosaic virus-[Congo:2008] (EACMV-[DRC:08])	FN435278	Cassava	Congo	[108]
East African cassava mosaic-Kenya [Tanzania:Dar Es Salaam:1996] (EACMV-KE[TZ:Dar:96])	Z83256	Cassava	Tanzania	[73]
East African cassava mosaic-Kenya [Tanzania:M] (EACMV-KE:[TZ:M])	AY795986	Cassava	Tanzania	[83]
East African cassava mosaic-Kenya [Tanzania:T] (EACMV-Ke[TZ:T])	AY795985	Cassava	Tanzania	[83]
East African cassava mosaic-Tanzania [Tanzania:YV] (EACMV-[TZ:YV])	AY795987	Cassava	Tanzania	[83]
East African cassava mosaic virus-Uganda-[Tanzania:10] (EACMV-UG[TZ:10])	AY795988	Cassava	Tanzania	[83]
*East African cassava mosaic Cameroon virus*				
East African cassava mosaic Cameroon virus-Tanzania[Tanzania:7:2001](EACMCV-[TZ:7:01])	AY795984	Cassava	Tanzania	[74]
*East African cassava mosaic Malawi virus*				
East African cassava mosaic Malawi virus-[Malawi:MH:1996] (EACMMV-[MW:MH:96])	AJ006459	Cassava	Malawi	[73]
East African cassava mosaic Malawi virus-[Malawi:K:1996] (EACMMV-[MW:K:96])	AJ006460	Cassava	Malawi	[73]
*East African cassava mosaic Zanzibar virus*				
East African cassava mosaic Zanzibar virus-[Tanzania:Uguja:1998] (EACMZV-[TZ:Ugu:98])	AF422174	Cassava	Zanzibar	[74]
*South African cassava mosaic virus*				
South African cassava mosaic virus-South Africa-[South Africa:99] (SACMV-[ZA:99])	AF155806	Cassava	South Africa	[76]
South African cassava mosaic virus-[Madagascar:12] (SACMV-[MG:12])	AJ422132	Cassava	Madagascar	[79]
South African cassava mosaic virus-[Zimbabwe:Muzarabani] (SACMV-[ZW:Muz])	AJ575560	Cassava	Zimbabwe	[80]
*Sweet potato mosaic associated virus*				
Sweet potato mosaic associated virus (SPMaV-[ZA:WP:2011])	JQ621843	Sweet potato	South Africa	[64]
*Sweet potato leaf curl Sao Paulo virus*				
Sweet potato leaf curl Sao Paulo virus (SPLCSPV-[ZA;WP:2011])	JQ621844	Sweet potato	South Africa	[64]
*Tobacco leaf curl Comoros virus*				
Tobacco leaf curl Comoros virus-[Grande Comore:Foubouni99:2005] (TbLCKMV-[GC:Fou99:05])	AM701762	Tobacco	Comoros	[63]
Tobacco leaf curl Comoros virus-[Grande Comore:Simboussa18:2004] (TbLCKMV-[GC:Sim18:04])	AM701760	Tobacco	Comoros	[63]
*Tobacco leaf curl Zimbabwe virus*				
Tobacco leaf curl Zimbabwe virus-[Zimbabwe:2001] (TbLCZV-[ZW:01])	AF350330	Tobacco	Zimbabwe	[78]
Tobacco leaf curl Zimbabwe virus-[Grande Comore:Foumboudziouni95:2005] (TbLCZV-[GC:Sim18:05])	AM701756	Tobacco	Comoros archipelago; Grande Comore	[63]
*Tomato curly stunt virus*				
Tomato curly stunt virus-[South Africa:Onderberg:1998](ToCSV-[ZA:Ond:98])	AF261885	Tomato	South Africa	[86]
Tomato curly stunt Lanseria virus*				
Tomato curly stunt Lanseria virus-[South Africa:Lanseria:2008] (ToCSLV-[ZA:Lan:08])	Access #	Tomato	South Africa	[145]
Tomato curly stunt Noordoewer virus*				
Tomato curly stunt Noordoewer virus-[South Africa:Noordoewer06:2008](ToCSNV-[ZA:Nwr06:08])	Access #	Tomato	South Africa	[145]
Tomato curly stunt Mooketsi virus*				
Tomato curly stunt Mooketsi virus-[South Africa:Mooketsi:2007] (ToCSMV-[ZA:Mks:07])	Access #	Tomato	South Africa	[145]
Tomato leaf curl Antsiranana virus*				
Tomato leaf curl Antsiranana virus-[Madagascar:Antsalaka6:2001] (ToLCAntV-[MG:Mia6:01])	AM701766	Tomato	Madagascar	[63]
Tomato leaf curl Antsiranana virus-[Madagascar:Miandrivazo1:2001] (ToLCTolV-[MG:Mia1:01])	AM701767	Tomato	Madagascar	[63]
*Tomato leaf curl Arusha virus*				
Tomato leaf curl Arusha virus-[Tanzania:Tengelu:2005] (ToLCArV-[TZ:Ten:05])	DQ519575	Tomato	Tanzania	[84]
*Tomato leaf curl Anjouan virus*				
Tomato leaf curl Anjouan virus-[Anjouan:Ouani3:2004] (ToLCAnjV-[Anj:Oua3:04])	AM701758	Tomato	Comoros archipelago; Anjouan	[63]
Tomato leaf curl Comoros virus*				
Tomato leaf curl Comoros virus-Mayotte:Dembeni:2003] (ToLCKMV-[YT:Dem:03])	AJ865341	Tomato	Comoros	[63]
Tomato leaf curl Diana virus*				
Tomato leaf curl Diana virus - [Madagascar:Namakely5:2001] (ToLCDiaV-[MG:Nam5:01])	AM701765	Tomato	Madagascar	[82]
*Tomato leaf curl Madagascar virus*				
Tomato leaf curl Madagascar virus-Androy [Madagascar:Toliary:2001] (ToLCMGV-And[MG:Tol:01])	AJ865339	Tomato	Madagascar	[82]
Tomato leaf curl Madagascar virus-Menabe [Madagascar:Morondova:2001] (ToLCMGV-Men[MG:Mor:01])	AJ865338	Tomato	Madagascar	[82]
*Tomato leaf curl Mayotte virus*				
Tomato leaf curl Mayotte virus-[Mayotte:Kahani:2003] (ToLCYTV-[YT:Kah:03])	AJ865340	Tomato	Comoros archipelago; Mayotte	[82]
*Tomato leaf curl Moheli virus*				
Tomato leaf curl Moheli virus-[Comoros:Fomboni163:2005] (ToLCMohV-[KM:Fom163:05])	AM701763	Tomato	Comoros archipelago; Grande Comore	[63]
Tomato leaf curl Namakely virus*				
Tomato leaf curl Namakely virus-[Comoros:Dimadjou:2001] (ToLCNaV:Dim:01)	AM701761	Tomato	Comoros archipelago; Dimadjou	[63]
ToLCNamV-[Madagascar:Namakely:2001] ([MG:Nam:01])	AM701764	Tomato	Madagascar	[63]
*Tomato leaf curl Seychelles virus*				
Tomato leaf curl Seychelles virus-[Mahe:Val d’Endor77:2004] (ToLCSV-[Mah:VE77:04])	AM491778	Tomato	Seychelles	[71]
Tomato leaf curl Tanzania virus-[Tanzania:1994]) (TYLCTZV-[TZ:94])	U734981523 bp	Tomato	Tanzania	[71]
Tomato leaf curl Toliara virus*				
Tomato leaf curl Toliara virus-[Madagascar:Miandrivazo2:2001] (ToLCTolV-[MG:Mia2:01])	AM701768	Tomato	Madagascar	[63]
*Tomato leaf curl Tanzania virus*				
Tomato leaf curl Tanzania virus (ToLCTZV)	U73498	Tomato	Tanzania	[71]
*Tomato yellow leaf curl virus*				
Tomato yellow leaf curl virus	HM448447	Tomato	Mauritius	[89]
*Defective subgenomic DNAs*				
*East African cassava mosaic virus* associated defective DNA-A (1,525 nt)		Cassava	Tanzania	[65]
TbLCZV-[ZW] associated ‘HG’ DI (1,341 nt)		Tobacco	Zimbabwe	[78]
TbLCZV-[ZW] associated ‘mild’ DI (1,421 nt)		Tobacco	Zimbabwe	[78]
SACMV-[ZA] associated defective DNA-B (1,389 nt)		Cassava	South Africa	[66]

* Viral isolates awaiting approval as species [10]; # not assigned yet.

#### 2.2.3. Evolutionary Relationships between SADC and SWIO Tomato-Infecting Begomoviruses

**Figure 3:**
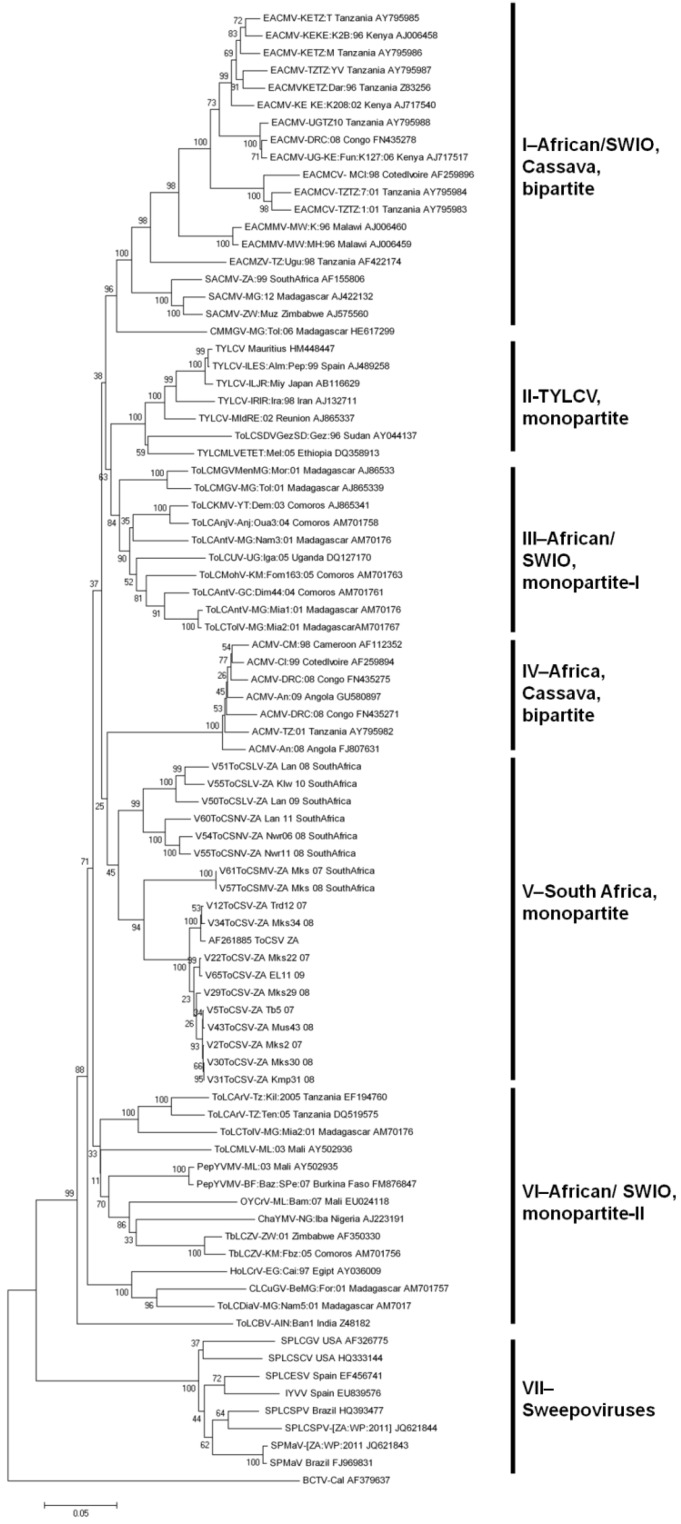
Neighbour joining phylogenetic tree indicating the relationship between the mono- and bipartite geminiviruses from southernSADC African countries and SWIO islands. Multiple sequence alignments were carried out using MUSCLE and phylogenetic analyses were performed using the neighbour-joining (NJ) and bootstrap option (1,000 replicates), available in Mega4.1. and Mega5.1. Horizontal branch lengths represent genetic distances as indicated by the scale bar, vertical distances are arbitrary. Representative begomovirus sequences obtained from Genbank are indicated by corresponding identifiers, numbers, abbreviations and accession numbers. The out-group is an isolate of *Beat curly top virus* (BCTV-B[US:Log:76]; AF379637).

The evolutionary relationships between all of these SADC and SWIO tomato infecting begomoviruses are depicted by the phylogenetic tree in Figure 3. The tomato infecting begomoviruses with some variation of the name ‘tomato yellow leaf curl virus’ are most closely related to one another and all cluster within the Mediterranean TYLCV-monopartite (II) clade. Besides the exotic TYLCV isolates from Mauritius and Reunion, none of these species has so far been found in the SADC/SWIO region. Viruses with some variation of the name ‘tomato leaf curl virus’ are more diverse and cluster within separate clades including many of the SADC and SWIO region tomato infecting begomoviruses. The phylogenetic clusters including tomato infecting viruses from these regions are the African/SWIO-monopartite-I group (clade III), the South African-monopartite group (clade V) and the African/SWIO-monopartite-II group (clade VI). The remaining clades (I and IV) contain the African and SWIO- region bipartite begomoviruses. Whereas the SWIO island tomato-infecting viruses mostly group within clade III (with their closest described mainland ancestor being *Tomato leaf curl Uganda virus*) or clade VI, those from South Africa group within clade V and are most closely related to bipartite viruses in the Africa/Cassava bipartite group (clade IV). The remaining African tomato-infecting isolates from Tanzania and Zimbabwe, cluster with central/west/north African (Mali, Burkina Faso, Nigeria and Egypt) and SWIO isolates (Madagascar and Comoros) in clade VI. The concordant phylogenetic and geographical clustering of South African and SWIO tomato-infecting viruses strongly suggests that they are all indigenous to their respective regions. This is further supported by the fact that recombination events detectable within the genomes of the South African and SWIO viruses display a marked tendency to have involved parental viruses from these same regions [63,81,82,88,145]. 

As is the case with cassava, the fact that tomatoes were introduced to the SADC/SWIO regions sometime after the sixteenth century implies, firstly, that all of the 18 known indigenous African tomato-infecting begomovirus species must have had (and probably continue to have) some natural indigenous African host species, and secondly, that over the intervening years all of these viruses have independently expanded their host ranges to include tomatoes. Curiously, this seemingly unlikely scenario has been played out multiple times in every region of the Old World where tomatoes have been introduced, an observation that likely reflects the possibility that tomatoes are unusually easy for Old World begomoviruses to infect [1,2,55,56,146].

#### 2.2.4. Emergence and Transmission

The lesson to be learned from the emergence of cassava and tomato-infecting begomoviruses throughout the Old World is that likelihood of emergence events is high when intensively cultivated exotic plant species are introduced into environments teeming both with indigenous begomoviruses and with whitefly types that will transmit these viruses between their natural hosts and the introduced plant species [7,25,56,147,148]. As the case with cassava, it is likely that the whitefly component of this equation has been key to the emergence of tomato-infecting begomoviruses in the SADC/SWIO regions [25,27]. Although information on vector populations in all the SADC and SWIO islands are unavailable, several indigenous *B. tabaci* types have been reported, in addition to the introduced B and Q type in a number of regions [39,40,42,43,81,82,149]. Most of these indigenous whitefly populations are capable of transmitting begomoviruses and may have played a role in begomovirus emergence. For example, the SSAF-5 type from South Africa and MS type reported from Tanzania and the neighbouring islands of Réunion, Mauritius, Seychelles and Madagascar, has been documented to colonize vegetable crops and vector tomato-infecting begomovirus species [43,81,82]. It is however the introduced types, rather than the indigenous types, that are expected to be the most important driver for begomovirus emergence [1,2,55,146]. The *B. tabaci* B type has thus far been reported in South Africa, Mozambique, Reunion and Mauritius [86,149,150] and the *B. tabaci* Q type in South Africa and Mozambique [43,150] and in all cases, their appearance has resulted in infection by new begomoviruses [89,144]. It is worthwhile pointing out however, that whereas the spread of the invasive B type of *B tabaci* throughout Meso and South America has seen the emergence in these regions of numerous tomato-infecting begomovirus, there have been no corresponding reports on the emergence in these regions of cassava infecting begomoviruses. These examples are particularly illustrative in that they highlight the importance of individual components of the “emergence” equation. Whereas begomovirus emergence events can occur even when the host species in question is indigenous (as is the case with tomatoes), they will also not necessarily occur in all host species following the invasive introduction of polyphagous vector types (as appears to be the case with cassava). Whereas both cassava and tomato are clearly susceptible to begomoviruses the crucial difference may be either that tomato is a particularly good “catch all” begomovirus host, or that tomatoes are far more intensively cultivated in Meso America and South America than is cassava. 

#### 2.2.5. Management and Control of Tomato-Infecting Begomoviruses

The management of begomoviral diseases around the world is mainly based on a combination of cultural practices, the insecticidal control of whitefly populations and the use of resistant tomato cultivars. Surveys aiming at quantifying the relative prevalence and efficacies of these control measures in the SADC and SWIO regions is lacking for all countries other than South Africa. ToCSV in South Africa has mainly been managed through the use of insecticide application, especially during the summer months when whitefly populations tend to expand most rapidly. However, commercial farmers in South Africa have also started using commercially available TYLCV tolerant or resistant cultivars which have been proven to better tolerate ToCSV infection than TYLCV susceptible cultivars [145,151]. Given that most farmers in the SADC and SWIO regions simply cannot afford to prevent losses to tomato-infecting begomovirus with insecticides, and that the development of resistant tomato cultivars seems possible, the integration of such resistance into the favoured open-pollinated cultivars grown throughout these regions would substantially mitigate the threat posed by begomoviruses.

The presence in the SADC and SWIO regions of invasive polyphagous *B. tabaci* types and thewidespread occurrence of large numbers of tomato-infecting begomovirus species is likely having a major, albeit currently unquantified, impact on tomato production throughout these regions. The sheer number and diversity of viruses that even relatively small localized surveys have found infecting tomatoes in SADC countries and on the SWIO islands suggests that there likely exist far more tomato infecting SADC/SWIO begomovirus species than have currently been described. There is therefore a pressing need for continued surveillance for and analysis of tomato plants displaying symptoms characteristic of begomovirus infections. Also, additional, surveys need to be carried out through these regions to identify the natural hosts from which these viruses have emerged. While such concerted virus sampling efforts would almost certainly yield a more comprehensive catalogue of SADC/SWIO tomato-infecting begomovirus diversity (something which is needed to inform the development of broadly begomovirus resistant tomato cultivars), they will also provide a valuable early warning system for both the emergence of new highly pathogenic variants of indigenous viruses and the presence of newly imported and potentially damaging exotic begomoviruses. An important lesson that must be learned from the global dissemination of TYLCV, is that human trafficking within these regions of both indigenous SADC/SWIO viruses and exotic tomato-infecting viruses from Europe and Asia must be prevented by whatever means possible. Although trafficking of these viruses by their natural whitefly vectors may be more difficult to control, it is likely that the widespread use of broadly begomovirus-resistant tomato cultivars may somewhat mitigate the negative impacts of such movements. 

### 2.3. Sweet Potato

#### 2.3.1. General Introduction

Sweet potato (*Ipomoea batatas* (L.) Lam.) is a dicotyledonous perennial plant, producing edible tuberous roots and belongs to the morning glory family, *Convolvulaceae*. Sweet potato is the only *Ipomoea* species of economic importance as a food crop [152]. It is the seventh most important staple crop and third most important root crop in the world [91,152]. Even though it originated in South/Central America, it is now cultivated and consumed in many tropical and sub-tropical regions, including several SADC countries such as South Africa, Zimbabwe, Malawi and Tanzania [152,153]. Wherever sweet potato is grown viruses are an important constraint on their production [85,154]. Depending on the cultivar, the infecting virus, the stage of infection and whether the crop is infected with a single or multiple viruses, plants may lose up to 100% of their yield [153,154]. Given the innate sensitivity of sweet potatoes to virus infection [154], multiple infections (often involving viruses in multiple different domains) are common and cause a generalised condition known as Sweet potato virus disease (SPVD). SPVD often arises as the result of a synergistic interaction between whitefly-transmitted criniviruses (family *Closteroviridae*) such as *sweet potato chlorotic stunt virus* (SPCSV), begomoviruses such as *Sweet potato leaf curl virus* (SPLCV), and aphid-transmitted potyviruses (family *Potyviridae*) such as *sweet potato feathery mottle virus* (SPFMV) [153,154].

SPVD was first described in 1940 in Uganda, Burundi, Rwanda and eastern Belgian Congo, but has now been reported from Tanzania, Zambia, Madagascar and, more recently, in South Africa [64,85,155,156,157,158,159]. The disease is characterized by chlorosis, small-deformed leaves, and severe stunting, and can reduce yields of infected plants by up to 80% [154] or 90% [158] making it the most economically important disease affecting sweet potatos in the SADC region [158]. The begomoviruses that infect sweet potatos are all monopartite, and are phylogenetically distinct from both the Old and New World begomovirus species and, as such, are generally known as ‘sweepoviruses’ [160]. In recent years, a number of sweepoviruses infecting *Ipomoea* species have been identified in several countries around the world [161,162,163,164,165,166,167,168,169,170,171,172,173], but to date, the only sweepoviruses infecting sweet potato in Africa have been reported from Kenya [164] and Uganda [165] and more recently in South Africa [64]. As with other begomoviruses, the sweepoviruses are naturally transmitted by *B. tabaci.* However, as with the CMBs that infect cassava, sweepoviruses are also transmitted via vegetative propagation [156]. Symptoms include chlorosis, leaf curling and malformation, and stunting (Figure 1e). 

#### 2.3.2. Recent Detection of Sweepoviruses in South Africa

As with other sweepoviruses, the South African isolates (the only SADC isolates that have been fully characterised genetically) have a typical monopartite genome organization and are very closely related to viruses characterised elsewhere in the New World. One was identified as a minor genetic variant of *Sweet potato mosaic associated virus* (SPMaV) [170] and the other as a novel strain of *Sweet potato leaf curl Sao Paulo virus* (SPLCSPV) [163]. Phylogenetic and recombination relationships of these isolates to other monopartite *Ipomoea*-infecting begomoviruses indicated that SPLCSPV-[ZA:WP:2011] was a natural recombinant of sweepoviruses consisting of two distinct parental genomic sequences from SPLCSPV and *Sweetpotato leaf curl Georgia virus* (SPLCGV). It is therefore likely that, as has been proposed for similar discoveries in other parts of the world such as in Sicily, Peru, US, China and India [168,169,170,171,172], these viruses are not indigenous to South Africa but are part of a global pool of viruses that is being circulated by the international movements of sweet potato germplasm. It is nevertheless interesting that the South African SPLCSPV isolate is apparently a natural recombinant of viruses resembling the Brazilian SPLCSPV isolate and a North American isolate of *Sweet potato leaf curl Georgia virus* [170]. This and other evidence of pervasive recombination amongst sweepoviruses strongly suggests that there exist very few, if any, geographical or biological barriers to completely free genetic exchange amongst these viruses. 

If the example of large sweet potato virus surveys in other parts of the world are generalisable [162,163,173], it is very likely that additional sweepoviruses will be discovered wherever surveys are carried out in sweet potato-growing SADC countries. The impact of these viruses on sweet potato yields, their uncontrolled global movements and the lack of resistant cultivars, implies that should an ultra-pathogenic variant of these viruses emerge from the genetic mixing pot that apparently exists, it could conceivably devastate sweet potato production globally. In this regard sweet potato growing countries in the SADC and SWIO regions (especially those where sweet potatos provide a substantial proportion of caloric intake) should perhaps take steps to both monitor the diversity of viruses that have already been introduced to these regions, and prevent the future importation of additional genetic variants. 

### 2.4. Tobacco

#### 2.4.1. General Introduction

Tobacco is cultivated globally, with an average annual production reaching 5.9 million tons of leaf in 1997, and expected to reach 7.1 million by 2010 [174]. The top producers of tobacco are China, India, Brazil and the United States, with an average production of 6.7 million tons of tobacco annually [174]. Tobacco is also grown extensively in many SADC and SWIO countries such as Madagascar, Mauritius, Malawi, Mozambique, South Africa, Swaziland, Tanzania and Zimbabwe: the last is the largest African producer of tobacco with an estimated 94,175 Ha/year harvested yielding 109,737 tons of leaf [91].

Tobacco leaf curl disease (TLCD) was first properly described in the Dutch East Indies in 1912, but may have been reported in southern Africa as early as 1902 [175]. Several reports of leaf curl symptoms were reported from Tanzania [176] and Zimbabwe [177], and the disease was shown to be transmitted by whitefly [178]. The symptoms of TLCD varied in the field from mild leaf curl and small leaf enations to vein thickening and severe leaf curl (Figure 1f), yellowing and sometimes cup‑shaped enations. It was only in 1981 that TLCD was associated with a geminivirus [179]. 

Because the symptoms of TLCD manifested differently in different geographical regions, and in different plant cultivars, it was realised even in the first half of last century that the disease likely had multiple causes [180,181]. In South Africa (SA), McClean (1940) characterized three different symptom phenotypes, and was able to transmit the disease using naturally occurring whitefly from the field. The whitefly species was later demonstrated to be *B. tabaci* [182], but all subsequent attempts to isolate a geminivirus failed. In a later study, [183], variation in symptom phenotypes were noted in tobacco cultivation areas in SA. More recently, differential disease phenotypes associated with TLCD in the Comoros archipelago have been attributed to a virus complex consisting of at least three distinct monopartite begomovirus species, with symptom expression varying depending on host and virus combinations [90]. While several species of tobacco-infecting geminiviruses (with the exception of one mastrevirus, all are begomoviruses) have been described from around the world [9], the relative contribution of these different species as causal agents of TLCD has not been elucidated. 

#### 2.4.2. Geographic Diversity of Tobacco Begomovirus Species

Given that, as with sweet potatoes, tomatoes and cassava, tobacco originated in the Americas it is interesting that like cassava, but unlike tomato, it does not seem to be particularly prone to infection by New World begomoviruses. In fact only one New World begomovirus species, *Tobacco leaf curl Cuba virus*, has ever been reported [184] whereas there are four known tobacco-infecting Old World begomovirus species [10]. The first tobacco-infecting geminivirus in the SADC region was isolated and characterized from Zimbabwe in 1997 [75]. Although only partial genome sequences were obtained for this virus it was clearly most closely related to, but likely a distinct species from a *Chayote mosaic virus* isolate from Nigeria and the virus was ultimately classified as *Tobacco leaf curl Zimbabwe virus* (TbLCZV) [78]. TbLCZV is only distantly related to *Tobacco leaf curl Japan virus* [185] and *Tobacco leaf curl Yunnan virus* [186] and it is very likely that, like the tomato and cassava infecting begomoviruses, it is an indigenous African species than has only merged as a tobacco pathogen since the introduction of tobacco to Africa in the 17th century. TbLCZVhas also been detected along with another distantly-related tobacco infecting virus species, *Tobacco leaf curl Comores virus* (TbLCKMV), in the Comoros archipelago [63]. TbLCKMV is a particularly interesting isolate in that it is most similar to one of the tomato-infection SWIO isolates mentioned earlier and appears to be part of a complex of SWIO indigenous viruses which all infect bean, tomato and tobacco on Madgascar, the Seychelles and the Comoros [63]. Crucially, it is evident that viruses in this 14+ species complex frequently recombine with one another implying that mixed infections between them are common. Although it is evident that TbLCZV is also recombinant, no sequences closely resembling the parents of this virus have ever been sampled (different parts of the TbLCZV genome are alternately most closely related to *Tomato leaf curl virus* and *Indian cassava mosaic virus*).

As with other begomoviruses very little is known about the natural host ranges of the TLCD‑causing begomoviruses. Small scale experimental host range studies amongst cultivated species have indicated that it is likely to be restricted to members of the family *Solanaceae* [75]. Further studies should aim to both characterize begomoviruses infecting plant species in the region and to carry out proper natural host range studies on viruses such as TbLCZV and TbLCKMV. Besides illuminating the likely natural “pre-emergence” niches of these viruses, such studies will identify the likely host-species that are the “mixing bowls” wherein begomovirus genetic exchanges most frequently occur. 

#### 2.4.3. Atypical Defective DNA Molecules Associated with TbLCZV from Zimbabwe

Despite the identification of several alpha- and beta satellites associated with TLCD in Asia [187] no TLCD associated beta-satellites were identified in the original study in Zimbabwe as beta-satellites sequences were only deposited in Genbank ten years later. Beta-satellites and have not been reported in tobacco in other SWIO and SADC regions, and since this situation is mirrored by the tomato‑infecting begomoviruses in these regions, it would suggest that some monopartite begomoviruses that are indigenous to these regions have evolved from an ancestral virus that either lost, or never gained, the need for a pathogenicity modulating satellite molecule. It is also possible, however, that many of these viruses are instead associated with currently undiscovered satellite molecules that are undetectable using the PCR-based protocols currently used for this purpose [188], and wider screening using rolling circle amplification (RCA) may reveal more beta-satellites or non‑beta satellite-like molecules.

Two unusual defective DNAs associated with TbLCZV-[ZW], namely ‘HG’ and ‘mild’ df DNAs, were detected in Zimbabwe tobacco cutlivar HG [78]. These were shown to be 1,341 nt and 1,421 nt, respectively, and showed interesting features [78]. The V1, V2 and C1 ORFs were all truncated, and df DNA ‘HG’ contained a 172 bp non-viral DNA-A region. While at the time of the study this non-virus sequence was never determined, we speculated that this may be of host plant origin, and possibly may contribute to the strange cup-shaped enations noticed in cv. HG plants infected with TbLCZV-[ZW] and df DNA. It is not unknown for begomovirus-generated subgenomic DNAs to contain fragments of host DNA, probably due to recombinational and rolling circle replication mechanisms and secondment of host DNA polymerase [189]. In our laboratory, we have isolated several subgenomic DNAs ranging from ±500–1,500 nt, from ToCSV-[ZA:Ond:98] infected tomato, and found some of these to contain tomato genomic DNA. The df DNAs isolated from infected tobacco were never shown experimentally to cause symptom amelioration of their cognate helper geminivirus [78]. However recently we blasted this 172 bp non-DNA fragment from the HG DI and found this to show 72% and 92% nucleotide sequence similarity with *Ageratum leaf curl Cameroon* and *Tomato leaf curl Togo* betasatellites, respectively (not published). This is exciting as it demonstrates recombination between defective DNA-A and beta-satellite sequences in tobacco, and supports the earlier comment that defective, beta-satellite and non beta-satellite-like molecules may be more widely distributed in the southern African region than previously suspected 

A perplexing feature of tobacco leaf curl since its earliest reports has been the observed variation in symptom phenotype and severity. McClean [184] described five forms of symptom phenotypes in South Africa, ranging from mild to severe, and different symptoms were also noted by Valand and Muniyappa (1992) [190] in India. Four different field tobacco samples collected in Zimbabwe in 1996 and 1997 [183] exhibited different symptoms: *N. tabacum* c*vs.* HG and Burley showed downward leaf curl and puckering, and vein thickening, while the leaves of the other two cultivars (unknown) showed either severe leaf curl (with cup-shaped enations) or mild upward leaf curling. Sequencing of the intergenic region (IR) and coat protein (CP) demonstrated that the four TbLCZV-[ZW] isolates were 98% similar [75]. Variation in symptoms were also observed with agroinoculation of tobacco with infectious TbLCV-[ZW] DNA-A clones [78], with *N. tabacum* cv. Samsun exhibiting symptoms more typical of the mild form B described by McClean [181], and *N. tabacum* cv.HG exhibiting mild form C symptoms [181]. The severe form of the disease (severe upward curling, vein thickenings and enations and severe leaf size reduction and distortion) was seldom observed in *N. tabacum*, but was common in agroinoculated *N. benthamiana*. Since the two df DNAs were associated with the “mild’ form of the disease, and no significant nucleotide differences (IR and CP) were noted between the four sequenced helper TbLCZV-[ZW] isolates, it was suggested that the df DNAs may be playing a role in symptom amelioration. However, the potential role of betasatellites in disease symptom modification, and the possibility of recombinant viruses contributing to the different leaf curl phenotypes cannot be ruled out. This was not considered at the time of the study, since the extent of recombination and betasatellites were not well known. Sequencing of full-length DNA-As from a large number of infected tobacco samples may reveal greater genetic diversity, and the search for a betasatellite may prove useful in elucidation of the aetiology of tobacco leaf curl disease in southern Afri*ca.*

### 2.5. Bean

French (green) beans (*Phaseolus vulgaris*) are grown by both small-scale farmers and commercial farmers in many SADC and SWIO countries including, Lesotho, Madagascar, Malawi, Mauritius, Swaziland, Tanzania, Zimbabwe and South Africa [91]. Amongst these South Africa is the biggest producer (3,000 Ha/year; 79,240 Hg/Ha/year) [91]. While a survey showed the presence of the potyvirus *Bean common mosaic virus* (BCMV) in many of these countries [191], no geminiviruses were reported until recently. Globally, several bipartite geminiviruses have been isolated from French beans, including *Bean golden mosaic virus* (BGMV) [192], *Bean dwarf mosaic virus* (BDMV) [193], and *Bean golden yellow mosaic virus* (BGYMV), all of which are New World begomovirus species. To date, the only geminivirus known to infect beans in the SADC region is the mastrevirus *Bean yellow dwarf virus* (BeYDV) first isolated in South Africa [72], which is most closely related (65% nt sequence identity) to *Tobacco yellow dwarf virus* (TbYDV)(from Australia) [194]. In the SWIO region a distinct strain of *Cotton leaf curl Gezira virus* (CLCuGV), a species originally found in cotton from Egypt and the Sudan, was reported to infect beans in Madagascar [63]. 

BeYDV can cause yield losses of between 85 and 92% in infected plants [72] with symptoms including brittle leathery leaves, with thickened and shortened internodes and downward curling of young leaves. Although the experimental host range of BeYDV includes *Nicotiana benthamiana*, *N. tabacum*, *Solanum lycopersicon*, *Datura stramonium* and *Arabidopsis thaliana* [72], it is again pertinent to point out that, as with the other viruses discussed here, the natural host range of BeYDV is unknown. It is also presently unknown where geographically BeYDV originated. The discovery that it is a non-divergent member of a legume-infecting mastrevirus species found outside South Africa and Sudan, exclusively in the vicinity of the Middle East and Pakistan, suggests that it may have been recently introduced from that region. The fact that both of the described South African BeYDV full genome sequences cluster within a much more diverse group of Pakistani chickpea and bean infecting sequences very strongly suggests that BeYDV has moved from Pakistan (or thereabouts) to Africa, and not *vice versa*.

Finally, it is interesting to note both that the cassava-infecting begomovirus SACMV is also able to infect *P. vulgaris* [76], and that EACMV and ACMV have been reported infecting uncultivated leguminous species such as *Centrosema pubescens* and *Puerariaavanica* in the DRC [108]. While this suggests that surveys of uncultivated legumes might reveal the natural host ranges of at least some of the cassava-infecting begomovirus species described above, it is also possible that these viruses might pose a future threat to cultivated legumes such as beans chickpeas. BeYDV is one of only three mastreviruses known to infect dicotyledenous hosts. Adaptation of monocot-infecting mastreviruses to dicotyledenous hosts was previously thought not to be widespread. However, in the past decade, as more geminivirsues have been discovered, it is evident that this may be a more frequent event. Evidence for this lies in the detection of some begomoviruses in weed, ornamental and alternate crops, such as cassava mosaic begomoviruses in the DRC, and in Nigeria [108,195], which may provide a melting pot of mixed geminivirus infections due to feeding by *Bemisiatabaci*, and facilitate survival of the viruses. An example is recombination among isolates of *Tobacco leaf curl Japan virus* and *Honey suckle yellow vein virus* from an ornamental plant [196]. Whiteflies may infect non-hosts where there is high epidemic pressure, and in intercropping agrosystems, adaptation may occur more frequently. Additional evidence for adaptation is the frequency of recombination that can occur in host and non‑host crops between monopartite and bipartite geminivirsues [118], previously discussed for ACMV under Section 2.1. Frequent mixed infections and recombination among begomoviruses are well known [25,28,60,61,197]. Re-introduction of geminiviruses back into host plants from alternate crops or weeds could potentially play a significant role in the epidemiology, not only of bean viruses, but in the emergence of new distinct geminiviruses.

## 3. Conclusions

Whitefly-transmitted geminiviruses are thought to have evolved in the Old World (Eastern Hemisphere), where both monopartite and bipartite viruses are extant [197]. The global movement of germplasm, and the efficient transmission by the cryptic species group of *B. tabaci*, which exhibits considerable genetic and biological variation, has resulted in the explosion of new begomoviral species complexes in many tropical, subtropical and mild temperate climes in the past three decades. Genetic recombination due to sharing of common hosts, and expansion of host ranges for some begomoviral species, has resulted in diverse species complexes which pose a serious threat to agriculture not only in southern and eastern African countries, but globally. While cassava mosaic disease-associated begomovirus diversity and complexes are the only ones to have been extensively studied in many regions of Africa [5,102], a recent study on emerging begomoviruses on bean, tobacco and tomato on the SWIO islands revealed unexpected high diversity (seven new monopartite viral species), and recombination events between African, Mediterranean and SWIO begomoviruses [63]. This was also noted in begomoviruses from southern Africa, namely TbLCZV, ToCSV and SACMV [76,78,114,145], where recombinant events were noted. As demonstrated with many other begomoviruses, recombination also appears to be the driving force in the diversity of begomoviruses in the southern, eastern African and SWIO regions. The introduction of exotic begomovirus species, for example sweet potato viruses from Latin America, and tomato leaf curl viruses from the Mediteranean, from non‑indigenous crops into the region, have contributed enormously to the emergence of begomovirus diversity, while the cassava begomoviruses appear to have recombined, although not exclusively, with gene fragments from viruses from indigenous crop species. TYLCV, on the other hand, as an exotic species has been introduced into Reunion and Mauritius [63], but there is no indication of recombination so far with local indigenous begomoviruses, but potentially could do so in the future. Surprisingly there have been comparatively few identified begomoviruses on a handful of eudicot crops, such as tomato, tobacco and bean, in these regions, as described above. Growing awareness of their economic impact, will most likely lead to wider surveys in crops as well as indigenous plant species and will reveal more virus species diversity and complexes. The recent and first discovery of two sweepoviruses in South Africa [64] is highly likely to be the result of infected vegetative germplasm introduced to South Africa due to the genetic relatedness to two Western Hemisphere begomoviruses, and emphasises the importance of rigorous screening to prevent the introduction of non-native begomoviral species into new environments. 

The emergence of cassava, tobacco and tomato-infecting begomoviruses throughout the SADC and SWIO regions was likely due to introduction and intensive cultivation of exotic crop species having been introduced into environments harbouring indigenous begomoviruses. Furthermore, the *B. tabaci* cryptic species complex (currently consisting of 24 distinct species and many haplotype variants) [38], that transmit these begomoviruses between their natural hosts and the introduced plant species, has likely played a significant role in the emergence of cassava, tobacco and tomato-infecting begomoviruses in the SADC/SWIO regions [25,27,28,43,77]. Given the propensity of begomoviruses to recombine, and frequent introduction of polyphagous whitefly types into novel regions and co‑adaptation to new cultivated crops, weeds and indigenous host plants, emergence of new recombinant begomoviruses with increased virulence can be predicted (expected) to occur. The emergence of several new tomato-infecting begomoviruses within the past 15 years in the SADC/SWIO regions [73,88,144,145] and several new cassava begomoviral species, including SACMV [76], demonstrate the frequent occurrence of recombination events. The implications for agricultural productivity and economic losses in the future are enormous, and more attention is needed to study begomovirus diversity and epidemiology in the region in order to be able to strategize disease management programs.
